# Lancing the Gums in Extraction

**Published:** 1865-09

**Authors:** Henry S. Chase

**Affiliations:** Iowa City, Iowa


### LANCING THE GUMS IN EXTRACTION.

BY HENRY S. CHASE, M. D., D. D. S., IOWA CITY, IOWA.

[Read before the Iowa State Dental Society.]

Considerable discussion has arisen lately in regard to the propriety of lancing the gums in the extraction of teeth. Some say that it is an entirely needless operation, productive of much pain, and indeed often exceeding that of extraction itself. People are apt to ride "hobbies." They take up an idea, dwell upon it, exaggerate its importance, and finally make themselves and their favorite theory ridiculous.

Now, it seems to me that those who *discard* the use of the lancet in the extraction of teeth, are open to the charge of "riding a hobby." It is very true that in many cases there is no need of lancing the gums; and it is equally true that in very many cases it *is* necessary to lance the gums, if we wish to perform a neat and skillful operation.

In extracting a tooth, or performing any other surgical operation, it is very proper to avoid giving unnecessary pain, and an operator who ignores this humane and christian sentiment, is an unworthy member of the profession which he disgraces. On the other hand, one who sacrifices any thing of real merit in the performance of a necessary operation, from undue sympathy with the sufferings of a patient, is also to be condemned.

I have for many years considered myself an "adept" in the extraction of teeth and roots. Probably every dentist of much experience has the same feeling of confidence in like abilities.

My practice has always been to use a *sharp* gum lancet whenever I thought a *better* operation could be performed with it than without it. In preparing a mouth for a set of artificial teeth, I seldom leave any portion of a root to be afterwards removed. A great many mouths have come under my observation, prepared by those who *never* (?) use the lancet; and I have almost invariably found several portions of roots to be removed, that had been left by those operators for future extraction. Their skill had been baffled, and Nature had been chosen to do their work. Now, I would not have it understood that I *invariably* remove *every* root at one sitting, or that I never call upon Nature for her assistance. I certainly do so when I think it for the best good of the patient, without any regard to those ideas of false professional pride, which might lead one to almost murder his patient, rather than have it said that he had failed in an attempt of extraction.

Doubtless the same generous motives influenced those who "prepared" these mouths. *But* I think if they had used the gum lancet judiciously, there would have been a less number of roots left in the mouth.

I am well aware that the necessity of lancing the gums for the extraction of *teeth with crowns*, is less frequent than formerly, owing to the improvement in the beaks of forceps, which are made thin and sharp, thereby dividing the tissues as they are pressed "home."

I consider it important in *every* case, that the beaks of the forceps should touch the alveolus of the tooth to be extracted, and in many cases should be made to embrace a portion of that bone itself. Particular cases will readily suggest themselves to the mind. For instance, where the *crown* is very frail, or absent, and the member decayed well down into the roots, nearly cutting the crown off. In ulcerating teeth, the alveolus is often absorbed, so that the instrument passes readily low down upon the roots without dividing tissues. When the gums are much inflamed about an aching tooth, the tissues are not unfrequently detached from the teeth and alveolus, so that no cutting is necessary.

But let me suppose a common case—one that we meet nearly every day. A patient calls to have a bicuspid *root* extracted. The gum nearly conceals the end of it. A small opening through the central portion of the gum enables you to "sound" the offending member. You find that it lays high up in the alveolus, and the lower portion is very much hollowed by decay. Shall we undertake to extract without dividing the tissues with a sharp lancet? *I* should not do so without it!

A great many similar cases, where the lancet is imperative, crowd upon my mind, and doubtless they as readily suggest themselves to your own, and I refrain from describing them.

I hope this Society will thoroughly discuss this question of lancing the gums, for some men of good reputation are writing as though they had discarded the operation. If I am behind the times in dental *improvement*, I should like to know it.

